# Systematic Meta-Analysis Identifies Co-Expressed Kinases and GPCRs in Ovarian Cancer Tissues Revealing a Potential for Targeted Kinase Inhibitor Delivery

**DOI:** 10.3390/pharmaceutics11090454

**Published:** 2019-09-02

**Authors:** Hugo Albrecht, Eric Kübler

**Affiliations:** 1 Drug Discovery and Development Group, Centre for Cancer Diagnostics and Therapeutics, Cancer Research Institute, University of South Australia, Adelaide 5000, Australia; 2 Institute for Chemistry and Bioanalytics, School of Life Sciences, University of Applied Sciences and Arts Northwestern Switzerland FHNW, 4132 Muttenz, Switzerland

**Keywords:** GPCR, kinase, cancer, targeted drug delivery, personalized medicine

## Abstract

The use of many anticancer drugs is problematic due to severe adverse effects. While the recent clinical launch of several kinase inhibitors led to tremendous progress, these targeted agents tend to be of non-specific nature within the kinase target class. Moreover, target mediated adverse effects limit the exploitation of some very promising kinase targets, including mitotic kinases. A future strategy will be the development of nanocarrier-based systems for the active delivery of kinase inhibitors using cancer specific surface receptors. The G-protein-coupled-receptors (GPCRs) represent the largest cell surface receptor family and some members are known to be frequently overexpressed in various cancer types. In the presented study, we used ovarian cancer tissues as an example to systematically identify concurrently overexpressed GPCRs and kinases. The rationale of this approach will guide the future design of nanoparticles, which will dock to GPCRs on cancer cells via specific ligands and deliver anticancer compounds after receptor mediated internalization. In addition to this, the approach is expected to be most effective by matching the inhibitor profiles of the delivered kinase inhibitors to the observed kinase gene expression profiles. We validated the suggested strategy in a meta-analysis, revealing overexpression of selected GPCRs and kinases in individual samples of a large ovarian cancer data set. The presented data demonstrate a large untapped potential for personalized cancer therapy using high-end targeted nanopharmaceuticals with kinase inhibitors.

## 1. Introduction

Globally, more than 140,000 ovarian cancer-related deaths and more than 200,000 new cases were reported in 2016, and this annual burden is expected to increase in the near future [1,2,3]. The five major histological ovarian cancer subtypes are high grade serous ovarian carcinoma (HGSOC), low grade serous ovarian carcinoma (LGSOC), low grade endometrioid carcinoma, clear cell ovarian carcinoma (CCOC) and mucinous carcinoma. Current treatment includes the application of platinum-based chemotherapy, poly(ADP-ribose) polymerase (PARP) inhibitors, MEK inhibitors as well as hormonal and immune therapies [4]. However, a major shortcoming of these and other drugs is poor bio-distribution leading to toxic side effects against healthy cells, thus narrowing the therapeutic window. This will be especially pronounced when applying a combination therapy in which various anti-cancer agents would have a synergistic negative effect on healthy cells. For example, it was reported that the simultaneous inhibition of several mitotic kinases may be toxic [5,6].

Recent years have seen rapid advances in the field of kinase inhibitor drugs. Imatinib was approved in 2001 as the first small molecule kinase inhibitor for cancer therapy. More than 30 novel kinase targeting drugs have been introduced to the clinic since then and many more are in various development phases [7,8]. Approximately 40 kinase targets are thought to be blocked by the kinase inhibitors launched to date or in clinical development [9]. To circumvent drug resistance and considering the large choice of kinase inhibitors acting on probably all known cancer pathways, future clinical research is very likely to move towards combination therapies, using engineered nanoparticles for the delivery of more than one kinase inhibitor at a time. Presently the majority of clinically available anti-cancer nano-formulations use passive targeting, exploiting the Enhanced Permeability and Retention Effect (EPR) [10]. In this case, passive diffusion through endothelial fenestrations of tumor tissue lead to a local build-up of nanoparticle concentrations, an effect further enhanced by the lack of efficient lymphatic drainage. However, nanoparticles also accumulate in various organs, mainly liver and spleen, by vascular escape through endothelial fenestrations [11]. Although side effects tend to be milder with kinase inhibitors than with cytotoxic drugs, many of these compounds associated with adverse effects such as myelosuppression, neuropathy and gastrointestinal damage. To minimize these effects, active targeting of functionalized drug conjugates to cancer cells via overexpressed receptors using receptor-specific ligands or antibodies shows promise [12,13]. This can both further enhance the anti-cancer potency on solid tumors and reduce toxic side effects on healthy cells, respectively. 

Tumor cells generally show a characteristic pattern of overexpressed membrane associated proteins such as receptors, transporters and adhesion molecules. G-protein-coupled-receptors are the largest family of trans-membrane receptors and some are known to be overexpressed in prevalent solid tumors. The most intensely studied targeting receptors from the GPCR family are the somatostatin [11,14,15,16], cholecystokinin [17,18], gastrin-releasing peptide (GRP) [19,20,21], lutein releasing hormone [22,23], and neurotensin receptors [24,25]. Considering the large number of known GPCR receptor family members, they appear to be under-represented in current research addressing active receptor targeting. We believe that many more GPCR ligands can be exploited to design ligand-drug conjugates or drug carriers that trigger receptor internalization and hence anti-cancer agent delivery directly into endosomal compartments from where they can reach the cytosol through endosomolytic procedures [26].

**Figure 1:**
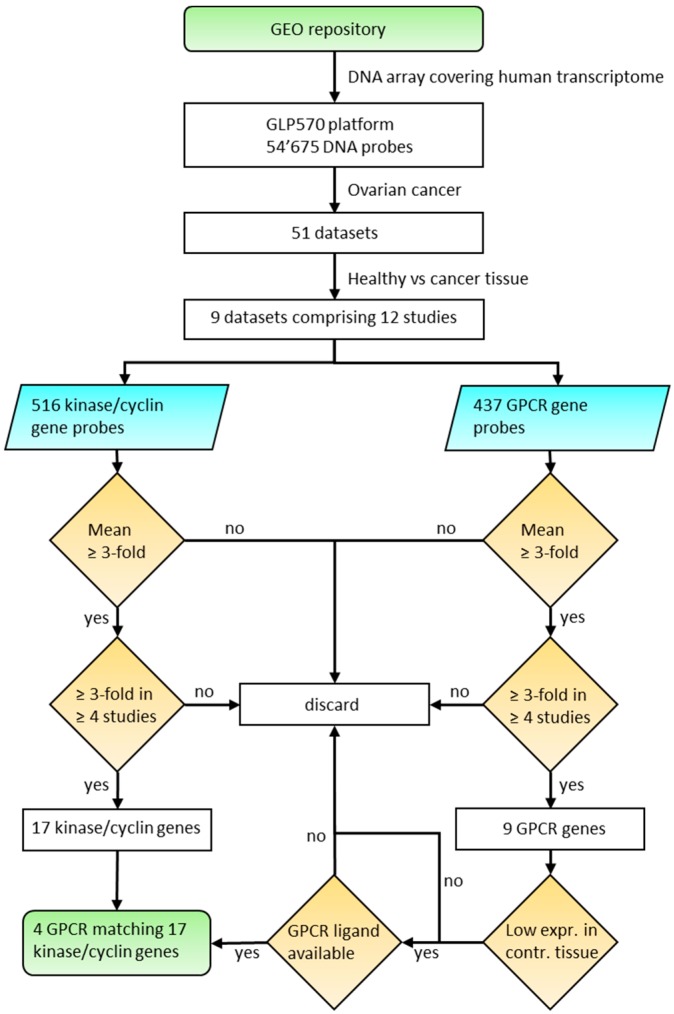
Algorithm to select overexpressed kinase/cyclin and GPCR genes in ovarian cancer tissue.

The aim of the presented study was to investigate the potential to actively target ovarian cancer cells using specifically engineered nanoparticles to deliver kinase inhibitors through overexpressed GPCRs. It is assumed that an anti-cancer strategy will be most effective through inhibition of overexpressed targets. Therefore, we systematically analyzed ovarian cancer tissues for concurrent high expression of GPCRs and kinases. We present meta-data from nine ovarian cancer microarray data sets with healthy tissues as controls compared to HGSOC, LGSOC, CCOC and tumor associated endothelial cells. Initially, 12 kinases and 5 cyclins (CCNs) were identified as strongly up-regulated. In the next step, the same data sets were systematically analyzed for overexpressed GPCRs, which led to an initial selection of nine receptors. The suitability of overexpressed receptors for specific cancer cell targeting with drug nanocarriers was assessed with respect to their expression levels in healthy tissues. The final selection contained four receptors (GPR39, LPAR3, OXTR and PTH2R) with strong expression in ovarian cancer tissue and generally low expression in human tissues. The logic of this approach is outlined in Figure 1. The expression levels of the selected genes obtained from an independent set of individual cancer tissue samples revealed high co-expression of receptors and kinases in most samples, albeit with a large heterogeneity particularly for GPCRs. These findings strongly support future potential for personalized medicine.

## 2. Materials and Methods

**Data set search strategy.** The NCBI GEO database was systematically searched to identify Entrez GEO DataSets with micro array expression data relevant for ovarian cancer. The search was focused on the GPL570 platform (single channel array) which represented >94% of all kinases, >90% of CNNs and >90% of non-olfactory GPCR genes. Only studies with information from primary cancer tissue were considered in our analysis. Hence, experiments with in vivo cell lines and xenograft models were excluded. Data sets with <20 samples and studies without control samples (e.g., healthy tissue) were not considered for the initial analysis of 9 data sets (Table 1). The primary selection of overexpressed genes from the nine data sets were compared to a large data set (GSE9891) which did not contain healthy tissue samples.

**Search for overexpressed GPCR, kinase and cyclin genes.** The CEL files of all datasets listed in Table 1 were retrieved from the NCBI GEO archive. The ArrayStar software (DNAStar Inc, Madison, WI, USA) was used to normalize the data with the robust multi-array average (RMA) [27] method to assure consistent handling of all data sets. Affymetrix annotation files were used to retrieve gene specific expression data with official gene symbols. The 7tm and GPCR Protein Family (Pfam) domains were used to identify all GPCRs listed in the Ensemble database (ensemble.org) and 755 GPCR genes were compiled including orphan, taste, olfactory and vomeronasal receptors. A subset of 437 receptors were represented by at least 1 DNA probe on the GPL570 platform. A list of 515 human kinase gene names was compiled from the UniProt database (https://www.uniprot.org/docs/pkinfam; Release 25 April 2018) and complemented with 32 cyclin gene names (including 2 CDK5 regulatory subunit genes) as retrieved from the HUGO Gene Nomenclature Committee (https://www.genenames.org/). The GPL570 platform contained 487 of the kinase and 29 of the cyclin genes.

**Statistical analysis.** Patient samples from different studies were stratified into groups of distinct subtypes and fold expression was calculated between cancer tissues and the corresponding normal tissues. An unpaired, two-tailed, equal variance Student’s t-test was applied to assess the significance of differentially expressed GPCR genes.

**Homology modeling and docking.** Receptor protein sequences were submitted to the Swiss-Model Server [28,29,30] and suitable templates were automatically searched in the SWISS-MODEL template library (SMTL, version 2017-10-23, last included PDB release 2017-10-13) using the Blast [31] and HHBlits [32] methods in parallel. The templates with the highest quality according to the global quality estimation score (GMQE) have then been selected for model building. Models were built based on the target-template alignment using ProMod3 and the global and per-residue model qualities were assessed using the QMEAN scoring function [33]. Finally, the CABS-dock server [34] was used for docking studies with selected peptide ligands against receptor models. The SwissDock server [35,36] was used for docking of lipid ligands. Various docking results were clustered according to the binding site and modality on the receptor. Visual checks of the structures were performed to discard clusters which showed binding in unexpected sites. Top ranked dockings from the finally prioritized clusters were rendered with Protean 3D (DNAStar Inc.) or Chimera (UCSF), and the structures were used to assess potential chemical conjugation for receptor targeting.

## 3. Results and Discussion

**Collection of gene expression data.** To select gene expression data sets allowing for the differential study of neoplastic ovarian cancer versus healthy tissue, the public GEO repository was systematically searched with a focus on a single DNA microarray platform to obtain easily compared data. For this purpose, the GPL570 human DNA array (Affymetrix Inc., Santa Clara, CA, USA) containing 54,675 DNA probes covering most of the human transcriptome was selected. The chosen platform represents >120,000 samples available from the GEO data base. A list of 515 kinase and 32 cyclin (CCN) genes was compiled (see supplementary info S1) and 487 kinase genes and 29 cyclin genes were identified on the GPL570 platform. All over, the chosen platform contained >94% of all kinases and >90% of all CCNs, which was considered suitable for the study (for detailed information on the lacking 28 kinase and 3 CCN genes, see supplementary info S1). An initial search for ovarian cancer data sets in the GEO repository limited to the GPL570 platform resulted in 51 hits. The data sets were checked for suitability according to the criteria outlined in the methods section and non-informative data sets were excluded. The final selection of nine ovarian cancer data sets is given in Table 1. 

**Figure 2:**
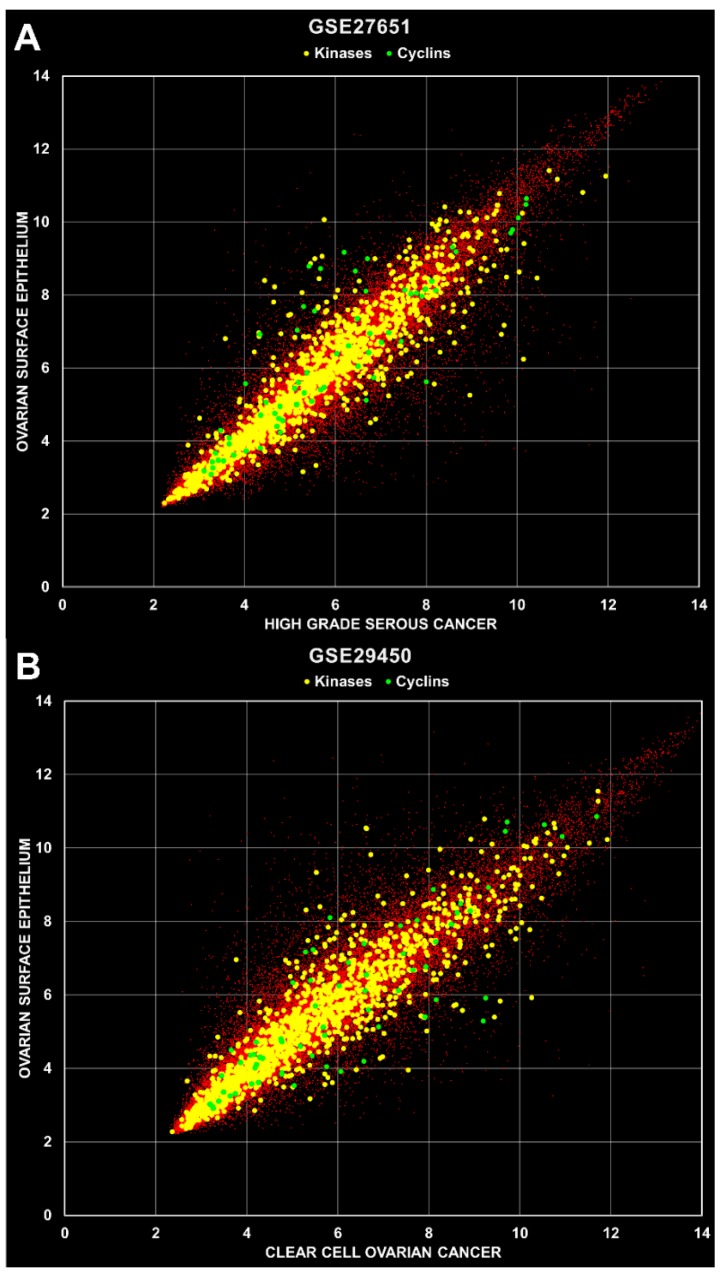
Scatter plots of microarray data. (**A**) Mean values were calculated from replica groups (HOSE 6 samples; HGSOC 22 samples) and plotted against each other as indicated with axis names. The *R*^2^ values were 0.8877, 0.8547 and 0.7522 for total, kinases and cyclins, respectively. (**B**) Mean values were calculated from 10 HOSE and 10 CCOC samples. The R^2^ values were 0.8577, 0.8406 and 0.7489 for total, kinases and cyclins, respectively.

**Identification of overexpressed kinases and cyclins in primary ovarian cancer tissue.** Cell data files were retrieved from the public GEO repository and processed using a robust multi-array (RMA) [27] normalization protocol with the ArrayStar software (DNAStar Inc, Madison, WI, USA). Annotations and attributes were imported automatically from files provided by Affymetrix Inc. (Santa Clara, CA, USA) and the data sets listed in Table 1 were organized into groups of replicates for expression analysis. Overexpressed kinases and cyclins were systematically identified by comparison of samples from cancer and healthy tissues, e.g., high grade serous ovarian carcinoma (HGSOC) versus human ovarian surface epithelium (HOSE) tissue. A total of 12 calculations were carried out with the nine data sets as indicated in Table 1, whereby the HG- and LGSOC, and the borderline ovarian tumor replicate groups were calculated separately for the GSE14001 and GSE27651 data sets. Two example scatterplots are depicted in Figure 2, where mean values of cancer versus healthy tissue replica groups are shown with kinase and cyclin genes indicated in yellow and green, respectively. All other genes are indicated with small red dots. 

With microarray data an induction of ≥2.0 fold is generally accepted as a good criterion for overexpression. Evaluation of our data revealed very robust differences and therefore all genes with an average of ≥3.0 fold induction in 12 calculations and ≥3.0 fold induction in four individual calculations were considered to be overexpressed. These criteria led to the selection of 12 kinase and 5 cyclin genes, which were overexpressed in 4 to10 calculations, about 7 on average (Table 2 and Table 3, respectively). A large proportion of these overexpressed genes play important roles mainly in mitosis (e.g., AURKA, BUB1, BUB1B, CDK1, MELK, NEK2, TTK, PBK) and cell growth/development (e.g., CDC7, ERBB3, PRKX, SYK) [37,38,39,40,41,42,43]. Inhibitors against some of these targets have been tested in clinical trials [44]. Noteworthy, AURK and CDK1 inhibitors showed anti-cancer efficacy in clinical trials but also induced strong adverse effects, including myelosuppression and gastrointestinal damage. This makes a very strong case for an approach aiming at specific delivery of these kinase inhibitors with engineered nanomedicines into cancer cells, while sparing healthy cells. Strongly expressed receptors can potentially be used for specific docking of ligand decorated drug carriers. For this purpose, we systematically searched for overexpressed GPCRs in ovarian cancer cells, which might offer an opportunity to develop such a strategy. 

**Some GPCRs showed massif overexpression in primary ovarian cancer tissue.** A systematic search for GPCR receptors that could serve as cellular attachment points for therapeutic kinase inhibitor formulations was performed using the same 12 calculations as outlined above. A list of 755 GPCR genes was compiled from 21 Pfam domains (7tm and GPCR) within the protein family database as reported previously [45] and 437 GPCR genes were identified on the GPL570 platform, which represented >90% of all non-olfactory GPCRs. Applying equal threshold characteristics as with the kinase and cyclin genes expression, 9 GPCR genes were identified with an average ≥3.0 fold expression in the 12 calculations and ≥3.0 fold in at least four individual calculations compared to healthy control tissues. Again, on average there was a ≥3.0 fold overexpression in about 7 of the 12 calculations per gene. A summary is given in Table 4. 

*p*-Values were calculated to assess differential expression of all selected GPCR, kinase and cyclin genes when cancer tissue was compared to normal tissue. The determined *p*-values were <0.1 in 77%, 75% and 66% for all kinase, CCN and GPCR calculations, respectively. Moreover, for sample groups with induction ≥3 fold, the *p*-values were ≤0.05 with only very few exceptions, namely three calculations (out of 97) for kinases and two calculations (out of 62) for GPCRs (Table 2 and Table 4). The fact that a significant amount of data sets did not show strong induction or in some cases showed even reduced expression, reflected the inherent heterogeneity of cancer samples. However, for two reasons we did not weigh the obtained inconsistencies too heavily in this study: (1) The selection of the GPCR, kinase and cyclin genes is based on data of a population of cancer samples and serves to narrow down possible target molecules (kinases and cyclins) and cellular entry sites (GPCRs). (2) while some of the selected GPCRs may be overexpressed in most cancer samples, others may merely be up-regulated in 30% or less of all cancer tissues. However, in the context of personalized cancer treatment, the consideration of these less frequently overexpressed receptors could still be of high practical value.

The next step was to select the most promising receptors for effective and safe cancer cell targeting. This is most likely achieved using receptors with low expression in healthy tissue and high expression in neoplastic tissue. The information for receptor expression in healthy tissues was collected from the Human Protein Atlas (HPA) and is shown in Table 5 with RNAseq data (RPKM; reads per kilo base per million mapped reads) given for each receptor in the lower row, and protein levels indicated in the upper row for each receptor, with 0 (not detected), 1 (low), 2 (medium) and 3 (high expression). 

**Figure 3:**
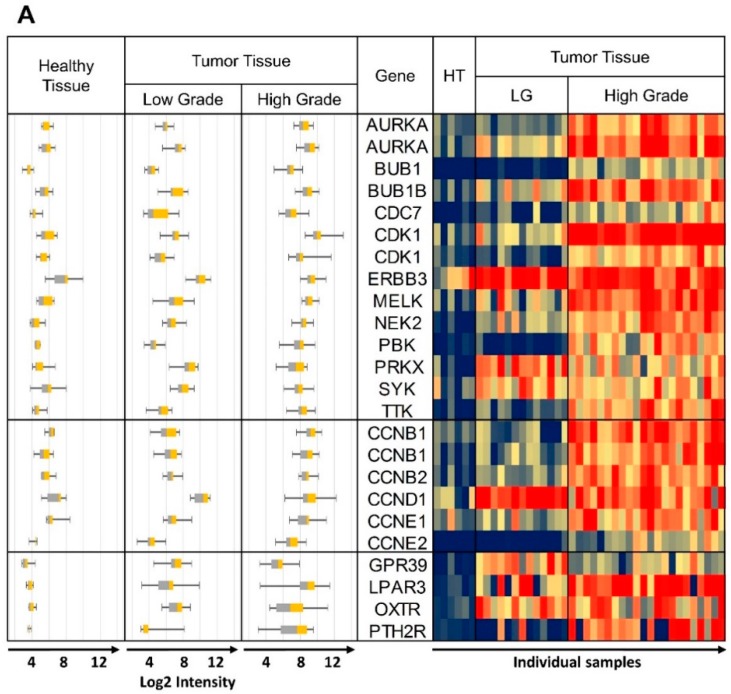

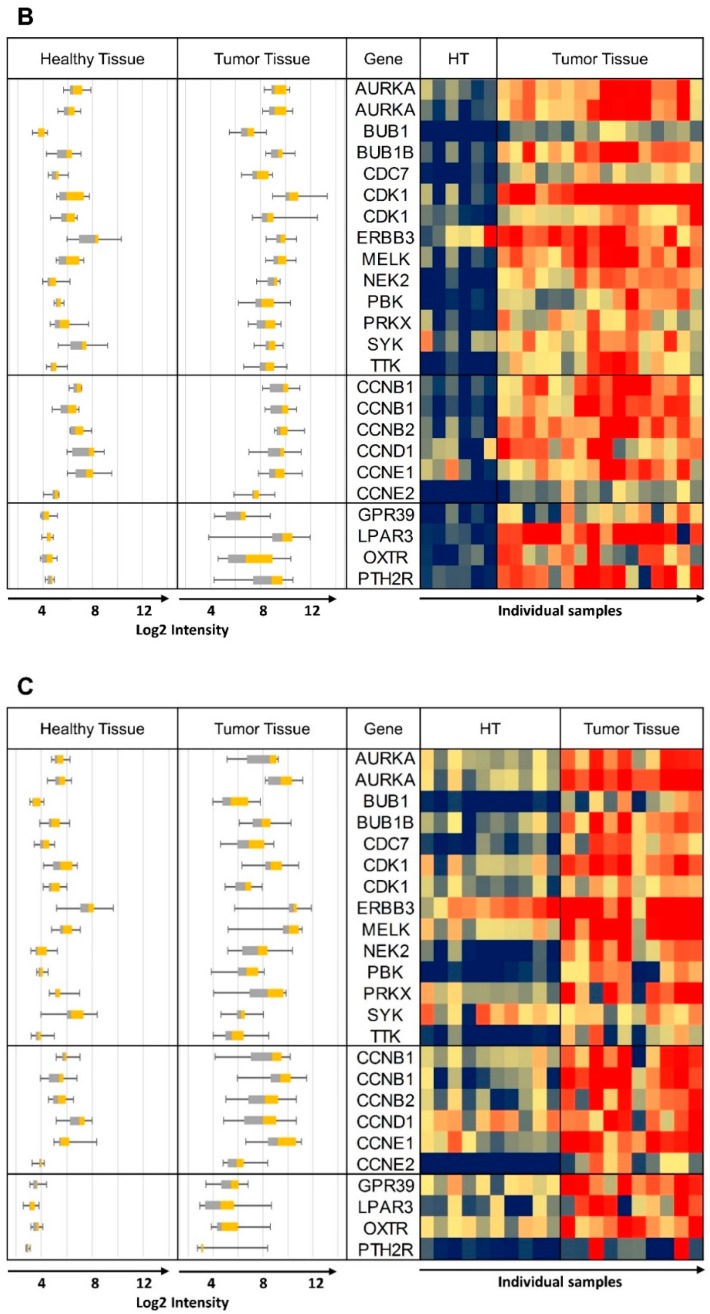
Heat maps of log 2 expression values from representative data sets. The same data are also shown as box plots on the left hand side of each panel. (**A**) GSE27651 Healthy Tissue (HT) vs. Low Grade (LG) and High Grade Tumor Tissue, (**B**) GSE54388 microdissection, Healthy Tissue (HT) vs. High Grade Tumor Tissue, (**C**) GSE29450 Healthy Tissue vs. CCOC. Whisker plots are shown on all panels: control healthy tissue left, tumor tissue right; heat map: control samples columns on left healthy tissue (HT), tumor tissue columns on right). Some of the kinase and CCN gene names are repeated due to more than one specific DNA probe included on the DNA microarray. Probe ID information can be obtained from Table 2, Table 3 and Table 4, with the duplicated gene names in the same top down order.

“Sum RNA” indicates the total sum of all RPKM values for individual receptors and similarly, “Sum Protein” is the total of all protein expression values. For safe cancer cell targeting, only receptors with low expression in healthy tissue were considered. An arbitrary cut-off at “Sum RNA” <100 and “Sum Protein” <50 revealed PTH2R, GPR39 (orphan receptor with known synthetic agonists), ADGRG2 (orphan receptor), OXTR and LPAR3 as the most promising candidates. From the orphan receptors ADGRG2 and GPR39, the former was discarded from the final selection due to lack of known agonists, while the latter was retained due to the recent discovery of synthetic agonists [46]. The next step was to analyze the co-expression of these receptors with candidate kinase targets in individual cancer samples. This was carried out first with samples from the GEO data sets listed in Table 1, and some results are presented in Figure 3 (for complete data see Supplementary Figure S2). The heat maps represent log2 expression levels and most data sets showed a clear differential expression of the selected genes between healthy and tumor tissue. All HGSOC (apart from GSE14401) and the CCOC tissue data sets showed overexpression for a majority of selected kinase/CCN and GPCR genes in tumor tissue. In the case of data set GSE27651 the overexpression was clearly stronger for HGSOC than for LGSOC (Figure 3A). All over, this analysis may indicate that the suggested strategy will be most suitable for HGSOC and CCOC. The samples for data set GSE54388 have been prepared by laser capture microdissection (Figure 3B) and differential expression of the selected genes seemed particularly pronounced. The deviating outcome with one data set (GSE14401) might be due to incomplete separation of tumor and healthy tissue during preparation procedures (Supplementary Figure S2). Future studies might deliver high quality data by applying laser capture microdissection to avoid non-cancer cells from confounding the experiments. An additional data set (GSE105437) was prepared with tumor associated endothelial cells and differential expression of the selected genes was not confirmed (Supplementary Figure S2). These results clearly demonstrate that our suggested strategy will be suitable to specifically select receptors to target ovarian cancer cells, but not the tumor vasculature. Finally, we conclude that (1) the expression between individual samples was heterogeneous to some degree and (2) most importantly, for most tissue samples, it will be possible to pick at least two strongly overexpressed GPCRs for specific targeting that can be matched with several overexpressed kinases and CCNs. 

**Figure 4:**
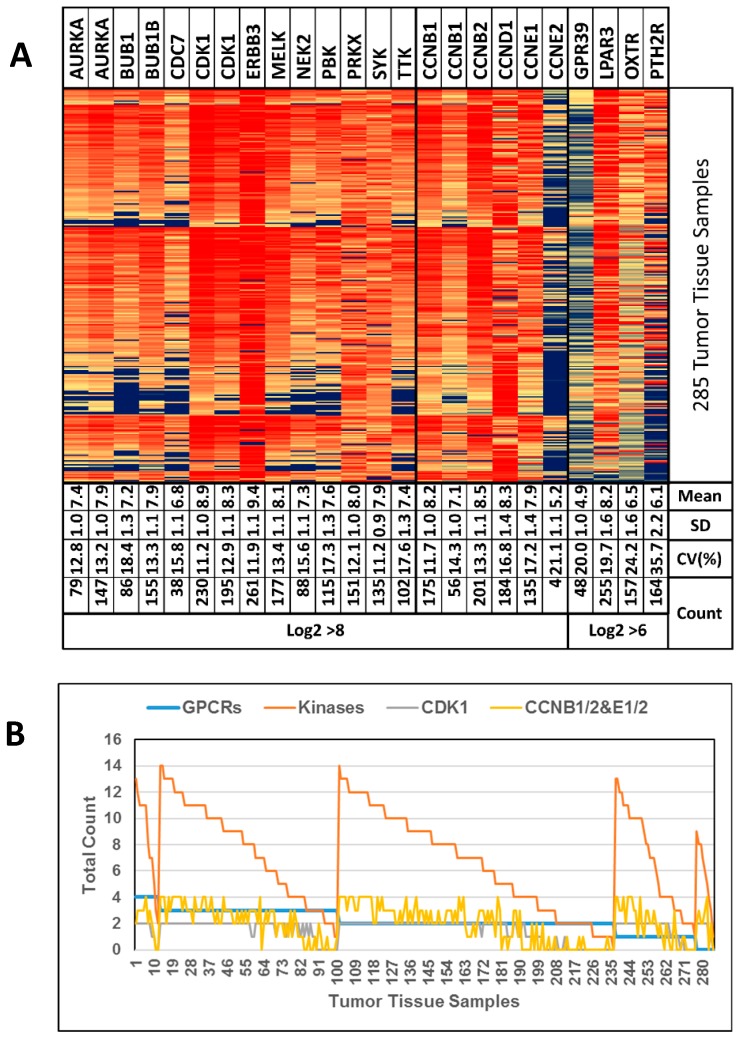
(**A**) Heat map of GSE9891 data. “Mean” values indicate Log2 intensities. “Count” values indicate the number of samples (out of 285 samples) with a Log2 intensity of >6 or >8, respectively. Genes AURKA, CDK1 and CCNB1 appear twice to show the data from 2 different DNA probes available. (**B**) Tumor tissue samples ordered according to the number of GPCRs with Log2 > 6 (blue line) followed by ordering them by the number of kinases with Log2 > 8 (orange line). CDK1 and CCNB1/2 are shown separately (grey and yellow line, respectively, see text for explanation). Genes with data from 2 DNA probes were looked at and counted individually.

To this point, we have analyzed mixed cancer tissue types including HGSOC, LGSOC, CCOC and cancer associated endothelial cells. We generated a series of heat maps and box plots for visual check and observed the strongest effects with HGSOC and CCOC samples. Subsequently we selected an independent large HGSOC data set (GSE9899) without negative controls to further consolidate our strategy. The obtained findings are visualized as a heat map in Figure 4A. The mean log2 expression levels were 7.9 for kinases and 7.5 for CCNs. The arbitrary cut-off for overexpression was set close to the mean at 8.0. Similarly for the GPCRs, which showed an average expression of 6.4, we chose the cut-off at 6.0. Strikingly, ERBB3 was strongly overexpressed in 92% of all cancer samples. Unfortunately this receptor has impaired kinase activity and therefore is not well suited for a small molecule kinase inhibitor strategy [47]. However it may be suitable for RNAi mediated down-regulation. Further analysis of the data revealed 7 kinases, 3 CCNs (including CCNB1 and B2), and 3 GPCRs, namely LPAR3, PTHR2 and OXTR to be overexpressed in at least 50% of all samples. Only nine samples did not show any overexpressed GPCRs and only one sample did not show any overexpressed kinase indicating that our strategy would be applicable for about 97% of HGSOC patients. 

Figure 4B depicts the number of GPCRs (maximally 4) and kinases (maximally 14) per individual sample that were overexpressed. CDK1 was the most frequently overexpressed kinase and is known to only be active as a dimer with CCNB1, 2, CCNE1 or 2 [48]. Therefore, we analyzed these genes separately and only 23 samples (8.1%) showed CDK1 overexpression without CCN overexpression (Figure 4B, grey and yellow line). 

GSE9891 was the largest HGSOC dataset from the initially identified 51 ovarian cancer data sets and was excluded from the initial analysis due to the lack of healthy control tissue samples.

In summary, the analysis with a large dataset further confirmed the potential to target most HGSOC cells with engineered nanocarriers, which display selected GPCR ligands on their surface for specific docking, followed by cellular internalization. Our results also strongly indicated the combination of the suggested future anticancer drug delivery strategies with the use of pan-kinase inhibitors, which match the gene expression profiles of cancer samples in a personalized manner.

**Figure 5:**
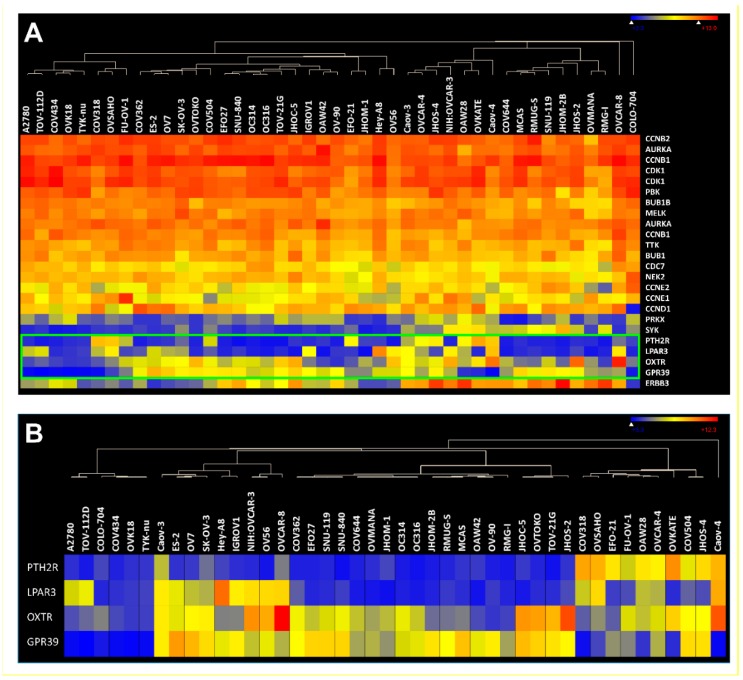
(**A**) Expression of 12 kinases, 5 CCNs and 4 final GPCRs from Table 2, Table 3 and Table 4 in 44 ovarian cancer cell lines (**B**) 4 final GPCRs clustered against 44 ovarian cancer cell lines.

**Co-expression of kinases/cyclins and GPCRs in human ovarian cancer cell lines.** To further support our findings, we looked at expression levels of the selected kinase/cyclin and GPCR genes in 44 ovarian cancer cell lines from the Cancer Cell Line Encyclopedia (CCLE) [49]. It became very apparent that based on the log2 absolute values most of the selected kinases and CCNs showed very strong expression in almost all cell lines (Figure 5A). In addition, the data confirmed that many receptors showed very high expression in cancer cells (Figure 5B). 

Since cancer cell lines are well accepted as clinically relevant research subjects, our results not only corroborate the results from the statistical expression analyses using primary tumor tissues but could also be the basis to generate in vitro models for the identification and characterization of new kinase inhibitors that can be specifically delivered through GPCR internalization. Finally, in vivo proof of concept could also be gained using these cell lines in xenograft models.

## 4. Ligand Docking

The knowledge of the relative orientation of ligand and receptor to each other is important for the choice of drug carrier–ligand conjugation sites. Additionally, the putative conjugation site on the ligand has to be exposed in a way that a drug carrier–ligand conjugation would not prevent binding to the receptor. Two of the four selected GPCRs and their respective ligands were chosen for docking experiments to gain structural insights in receptor–ligand complexes in order to reckon their amenability to function in targeted therapeutics. The PTH2 receptor primary amino acid sequence was taken as input to build homology-based models using the Swiss-Model Server [28,30]. In a next step, the highest scoring models based on the QMEAN scoring function was forwarded to the public CABS-dock server with which docking studies with the “tuberoinfundibular peptide of 39 residues” (TIP39) peptide ligand were performed. The LPAR3 amino acid sequence was treated similarly but there the docking studies were performed using the publicly available SwissDock server with the lysophosphatidic acid ligand. 

**Figure 6:**
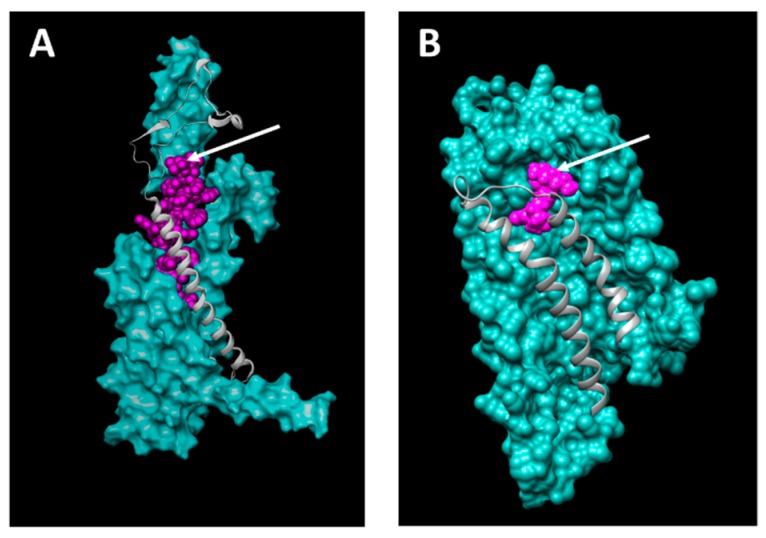
Docking of TIP39 and lysophosphatidic acid (LPA) to PTH2R (**A**) and LPAR3 (**B**), respectively. Arrows indicate potential anti-cancer drug carrier conjugation sites on the C-terminal carboxy group of TIP39 (**A**) and the *cis*-double bond of the aliphatic chain of LPA (**B**).

TIP39 is known to be a very potent and specific PTH2R agonist, while it only exerts weak antagonistic effects on the widely expressed PTH1R [50]. For this purpose it will be an optimal ligand to target PTH2R overexpressing ovarian cancer cells while sparing PTH1R expressing healthy cells. Figure 6 shows that the relatively large TIP39 ligand exposes its C-terminus (arrow) such that a drug carrier might be conjugated to it. Figure 6B shows lysophosphatidic acid (LPA) bound to LPAR3, and it seems most promising to chemically conjugate a drug carrier to the *cis*-double bond or at the omega end. LPA is a non-specific agonist of all six LPA receptors. Some of them are widely expressed in various tissues and therefore we suggest using the LPAR3-specific LPA derivative 1-oleoyl-2-methyl-sn-glycero-3-phosphothionate (OMPT) as ovarian cancer targeting ligand. OMPT only shows minor structural changes and is assumed to dock similarly to the target receptor.

## 5. Conclusions

**Figure 7:**
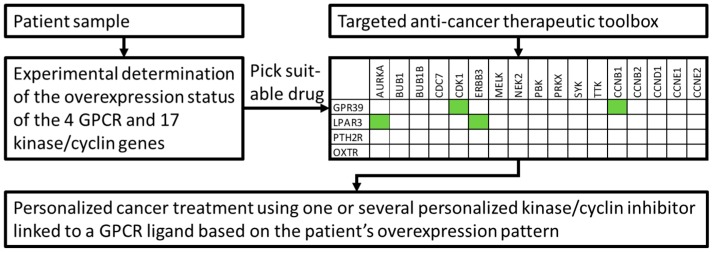
Example of a cancer patient’s molecular diagnostics leading to personalized and targeted cancer treatment. Green boxes depict four possible “kinase/cyclin inhibitors—GPCR entrance site” combinations based on patient’s overexpression data.

Cancer is a highly heterogeneous disease and GPCR overexpression (as well as other protein levels) may vary significantly among the different cancer forms. In addition, cancer patients exhibit a diverse phenotype even when suffering from the same type of cancer. Consequently, there is probably no greater niche to apply personalized therapeutics as in cancer treatments. However, such novel treatment paradigms will involve increasingly complex combinations of various drug substances. The delivery of such therapeutics via engineered nanoparticles will undoubtedly occur in the near future leading to highly efficacious and personalized cancer eradication. Experimental determination of both the cancer cell specific entry site (e.g., GPCRs) and the therapeutic target (e.g., kinases) would perfectly well fit into such an approach. The size of the sectional area of the overexpressed GPCRs and overexpressed kinases originating from our statistical analyses support this idea. Confirmation came from an analysis of an independent data set showing that 275 of 285 investigated individual cancer samples overexpressed at least one GPCR/kinase pair. It is now possible to conceive a toolbox for personalized medicine that consists of ligands to all 4 selected GPCRs each coupled to a nanomedicine inhibiting one or several of the 12 kinases (Figure 7). After testing a cancer patient’s personal GPCR/kinase/cyclin expression profile, one or several of the GPCR-ligand/nanomedicine conjugates can be considered to be therapeutics.

## Figures and Tables

**Table 1:** Gene expression datasets.

Accession #	Sample Size	Primary Cancer Samples	Calculations ^a^
GSE10971	37	Non-malignant fallopian epithelium (12 BRCA wt; 12 BRCA mut ^b^) versus high grade SOC ^c^ (13)	1
GSE14401	23	HOSE (3) ^d^, low grade SOC (10), high grade SOC (10)	2
GSE14407	24	HOSE (12), high grade SOC (12)	1
GSE18520	63	Normal ovary (10), advanced stage high grade SOC (53)	1
GSE27651	49	HOSE (6), serous borderline ovarian tumors (8), low grade SOC (13), high grade SOC (22)	3
GSE29450	20	HOSE (10) versus clear cell ovarian carcinoma (10)	1
GSE52037	20	Healthy (10) versus primary tumors (10) ^e,f^	1
GSE54388	22	Healthy (6) versus high grade SOC ^f^ (16)	1
GSE105437	22	Normal tissue (5), cancer (10) ^g^, [wound (7)] ^b^	1

^a^ Number of cancer replicate sets compared to healthy tissue replicates; ^b^ Not used in this study; ^c^ serous ovarian carcinoma; ^d^ Human ovarian surface epithelium; ^e^ Serous papillary adenocarcinoma; ^f^ Laser capture microdissected; ^g^ Tumor associated endothelial cells.

**Table 2:** Overexpressed kinases in ovarian cancer tissue detected by GPL570 microarray. Selected genes showed mean fold induction of ≥3.0, and ≥3.0 fold induction in at least four independent calculations (indicated in bold); *p*-values are indicated in brackets with 0.00 < 0.005.

Gene	Probe ID	Fold Induction (*p*-Value)
AURKA	204092_s_at	**13.35** (0.00), **10.85** (0.00), 0.44 (0.09), 0.78 (0.64), 2.75 (0.00), **5.46** (0.00), **7.04** (0.00), 1.19 (0.51), **5.71** (0.00), 2.64 (0.00), **6.3** (0.00), 1.4 (0.55); **mean: 4.83**
	208079_s_at	**15.46** (0.00), **11.82** (0.00), 0.19 (0.02), 0.62 (0.49), **5.69** (0.00), **6.31** (0.00), **10.27** (0.00), 3.00 (0.00), **16.29** (0.00), **5.25** (0.00), **9.24** (0.00), 1.44 (0.58); **mean: 7.13**
BUB1	209642_at	**6.24** (0.00), **5.99** (0.00), 0.33 (0.1), 0.73 (0.66), 2.71 (0.00), **6.20** (0.00), **9.43** (0.00), 1.59 (0.04), **4.41** (0.00), **3.36** (0.00), **9.36** (0.00), 1.53 (0.35); **mean: 4.32**
BUB1B	203755_at	**10.9** (0.00), **13.95** (0.00), 0.35 (0.05), 1.05 (0.93), **5.62** (0.00), **7.00** (0.00), **10.7** (0.00), **3.02** (0.01), **7.59** (0.00), **6.08** (0.00), **12.09** (0.00), 1.95 (0.32); **mean: 6.69**
CDC7	204510_at	**6.69** (0.00), **6.24** (0.00), 0.45 (0.03), 0.99 (0.98), 1.90 (0.01), 2.77 (0.00), **6.31** (0.00), 1.64 (0.19), **6.25** (0.00), 1.66 (0.05), **6.68** (0.00), 0.80 (0.74); **mean: 3.53**
CDK1	203213_at	**7.03** (0.00), **6.28** (0.00), 0.34 (0.05), 0.93 (0.90), **4.25** (0.00), **8.72** (0.00), **20.02** (0.00), 2.46 (0.04), **8.86** (0.00), **4.31** (0.00), **18.85** (0.00), 2.25 (0.28); **mean: 7.02**
	210559_s_at	**7.86** (0.00), **6.17** (0.00), 0.32 (0.01), 0.77 (0.54), 2.72 (0.01), **5.69** (0.00), **7.01** (0.00), 1.01 (0.97), 2.77 (0.00), 2.21 (0.05), **7.22** (0.00), 1.44 (0.56); **mean: 3.77**
ERBB3	226213_at	0.86 (0.84), 0.98 (0.98), **6.28** (0.00), **7.83** (0.00), 1.11 (0.77), 2.05 (0.04), **3.22** (0.06), **4.85** (0.02), **5.19** (0.02), 1.03 (0.92), **3.20** (0.08), 1.31 (0.63); **mean: 3.16**
MELK	204825_at	**6.19** (0.00), **5.44** (0.00), 0.18 (0.00), 0.47 (0.06), **6.84** (0.00), **5.86** (0.00), **11.01** (0.00), 2.56 (0.06), **13.18** (0.00), **5.32** (0.00), **10.77** (0.00), **3.59** (0.13); **mean: 5.95**
NEK2	204641_at	**3.50** (0.02), 2.81 (0.02), 0.66 (0.5), 1.38 (0.62), **5.43** (0.00), **11.06** (0.00), **15.65** (0.00), **4.96** (0.00), **12.01** (0.00), **6.59** (0.00), **17.27** (0.00), 2.59 (0.03); **mean: 6.99**
PBK	219148_at	**4.04** (0.00), **4.81** (0.00), 0.14 (0.00), 0.40 (0.16), 2.52 (0.04), **4.23** (0.00), **9.03** (0.00), 0.92 (0.69), **5.38** (0.00), 2.76 (0.03), **7.56** (0.00), 2.05 (0.29); **mean: 3.65**
PRKX	204061_at	0.67 (0.34), 0.69 (0.29), **5.56** (0.00), **3.28** (0.00), 1.39 (0.13), **4.50** (0.00), **5.59** (0.00), **11.54** (0.00), **5.24** (0.02), 1.62 (0.07), **6.23** (0.00), 0.66 (0.23); **mean: 3.91**
SYK	226068_at	1.15 (0.77), 1.25 (0.68), **6.55** (0.00), **6.17** (0.00), 2.41 (0.02), 1.6 (0.19), **4.04** (0.01), **4.52** (0.01), 0.91 (0.86), 2.43 (0.01), **3.19** (0.05), 1.97 (0.12); **mean: 3.02**
TTK	204822_at	**7.22** (0.00), **8.01** (0.00), 0.24 (0.03), 1.06 (0.94), **4.87** (0.00), **8.68** (0.00), **11.88** (0.00), 1.74 (0.09), **3.61** (0.01), **3.46** (0.02), **12.12** (0.00), 1.68 (0.31); **mean: 5.38**

**Table 3:** Overexpressed cyclins in ovarian cancer tissue detected by GPL570 microarray. Selected genes showed mean fold induction of ≥3.0, and ≥3.0 fold induction in at least four independent calculations (indicated in bold); *p*-values are indicated in brackets with 0.00 < 0.005.

Gene	Probe ID	Fold Induction (*p*-Value)
CCNB1	214710_s_at	**5.63** (0.00), **8.42** (0.00), 0.13 (0.00), 0.35 (0.1), 2.2 (0.05), **4.18** (0.00), **7.91** (0.00), 0.94 (0.88), **4.86** (0.01), 1.82 (0.14), **7.63** (0.00), 1.83 (0.38); **mean: 3.83**
	228729_at	**4.94** (0.00), **6.22** (0.00), 0.20 (0.01), 0.51 (0.26), **3.18** (0.01), **5.16** (0.00), **10.37** (0.00), 2.06 (0.07), **14.81** (0.00), **3.15** (0.01), **12.16** (0.00), 1.28 (0.68); **mean: 5.34**
CCNB2	202705_at	**11.87** (0.00), **10.79** (0.00), 0.43 (0.09), 0.94 (0.92), **4.85** (0.00), **5.69** (0.00), **8.34** (0.00), 1.96 (0.04), **5.65** (0.01), 3.60 (0.00), **8.33** (0.00), 1.89 (0.25); **mean: 5.36**
CCND1	208712_at	0.59 (0.19), 0.61 (0.25), 0.87 (0.67), 0.89 (0.80), 2.32 (0.06), **3.17** (0.00), **4.92** (0.00), **10.58** (0.00), 2.22 (0.2), **3.84** (0.00), **3.85** (0.02), 2.77 (0.13); **mean: 3.05**
CCNE1	213523_at	**18.67** (0.00), **13.54** (0.00), 0.48 (0.00), 1.17 (0.59), **4.82** (0.00), **5.74** (0.00), **4.66** (0.00), 1.32 (0.54), **10.03** (0.00), **4.71** (0.00), **3.63** (0.03), 1.83 (0.00); **mean: 5.88**
CCNE2	205034_at	**6.57** (0.00), **6.15** (0.00), 0.16 (0.00), 0.27 (0.01), 2.8 (0.00), **3.53** (0.00), **6.03** (0.00), 0.95 (0.84), **4.41** (0.00), 1.63 (0.14), **6.7** (0.00), 1.24 (0.62); **mean: 3.37**

**Table 4:** Overexpressed GPCRs in ovarian cancer tissue detected by GPL570 microarray. Selected genes showed mean fold induction of ≥3.0, and ≥3.0 fold induction in at least four independent calculations (indicated in bold); *p*-values are indicated in brackets with 0.00 < 0.005.

Gene	Probe ID	Fold Induction (*p*-Value); Mean Fold Induction
ADGRG1	212070_at	1.27 (0.31), 1.07 (0.84), **3.32** (0.16), 2.73 (0.26), **4.62** (0.00), **5.70** (0.00), **9.08** (0.00), **17.23** (0.00), **9.29** (0.00), **5.22** (0.00), **6.81** (0.00), 1.58 (0.43); **mean: 5.66**
ADGRG2	206002_at	1.16 (0.89), 1.66 (0.57), **16.73** (0.00), **7.60** (0.00), 0.57 (0.33), 1.59 (0.16), **4.64** (0.01), **16.58** (0.00), 0.25 (0.01), 1.22 (0.73), **4.05** (0.05), 1.14 (0.71); **mean: 4.77**
CXCR4	217028_at	0.80 (0.32), 0.73 (0.12), **18.04** (0.00), **42.93** (0.00), **4.31** (0.05), **3.26** (0.00), **9.41** (0.00), **6.32** (0.01), **7.45** (0.00), **5.28** (0.04), **8.52** (0.00), **7.53** (0.12); **mean: 9.55**
GABBR1, UBD	205890_s_at	**31.64** (0.00), **34.43** (0.00), **5.13** (0.00), **3.68** (0.00), 1.28 (0.63), 1.26 (0.45), 2.13 (0.19), 1.11 (0.87), 1.92 (0.4), 1.18 (0.74), 2.00 (0.32), 2.31 (0.06); **mean: 7.34**
GPR39	229105_at	1.00 (1.00), 1.21 (0.67), 0.26 (0.00), 0.20 (0.00), 2.44 (0.02), **3.19** (0.00), **4.06** (0.00), **13.10** (0.00), **3.36** (0.00), **3.52** (0.00), **3.88** (0.00), 1.40 (0.33); **mean: 3.13**
LGR6	227819_at	1.19 (0.84), 1.14 (0.88), **10.31** (0.00), **6.02** (0.00), 2.64 (0.01), **4.57** (0.00), **8.18** (0.00), **26.42** (0.00), 1.16 (0.68), **3.52** (0.00), **5.82** (0.00), 0.52 (0.27); **mean: 5.96**
LPAR3	231192_at	0.17 (0.00), 0.23 (0.02), **3.17** (0.00), **14.73** (0.00), **3.00** (0.11), **19.72** (0.00), **30.52** (0.00), **5.29** (0.01), **3.02** (0.04), **3.45** (0.04), **30.62** (0.00), 1.81 (0.28); **mean: 9.64**
OXTR	206825_at	1.76 (0.39), 1.20 (0.79), 0.10 (0.00), 0.10 (0.00), 1.44 (0.20), **5.04** (0.00), **7.78** (0.00), **9.43** (0.00), **3.10** (0.01), 1.56 (0.08), **6.56** (0.00), 1.88 (0.17); **mean: 3.33**
PTH2R	206772_at	**7.23** (0.01), **6.84** (0.01), 1.47 (0.01), **4.74** (0.00), **4.02** (0.02), **8.28** (0.00), **9.94** (0.00), 1.36 (0.46), 1.94 (0.23), **3.76** (0.04), **13.39** (0.00), 1.43 (0.38); **mean: 5.37**

**Table 5:** Protein and mRNA expression in healthy tissue. Data were collected from the Human Protein Atlas (HPA). Protein expression level (P) is indicated with 0, 1, 2 or 3 (dark green, light green, orange and red, respectively) representing no, low, medium or high expression, respectively. mRNA expression (R) is shown as reads per kilo base per million mapped reads (RPKM; dark green representing 0, light green 1–3, orange 4–9 and red >9 RPKM). White boxes: No protein or RNA expression data available from HPA.

GeneSymbol	Protein/RNA	CXCR4	ADGRG1	LPAR3	PTH2R	LGR6	GPR39	ADGRG2	OXTR	GABBR1	GeneSymbol	Protein/RNA	CXCR4	ADGRG1	LPAR3	PTH2R	LGR6	GPR39	ADGRG2	OXTR	GABBR1
Pituitary Gland	P										Oral Mucosa	P		2		0	1	0	0		0
	R	20	6	0	0	11	0	1	0	33		R									
Hypothalamus	P										Esophagus	P		2		0	1	1	0		0
	R	8	18	1	1	3	0	0	3	72		R	9	15	4	0	3	0	0	1	13
Cerebral Cortex	P		1		1	2	1	0		2	Stomach	P		2		1	1	0	0		0
	R	3	21	2	2	0	0	0	1	88		R	14	14	0	0	0	2	2	0	17
Hippocampus	P		0		0	1	2	0		2	Duodenum	P		3		0	3	0	0		0
	R	6	16	2	1	0	0	0	1	56		R									
Caudate	P		0		0	0	3	0		1	Small Intestine	P		3		0	2	0	0		0
	R	5	22	1	0	0	0	0	3	91		R	151	6	0	0	1	1	1	0	19
Cerebellum	P		0		0	0	1	0		2	Colon	P		3		1	3	0	0		0
	R	1	6	0	0	3	0	0	1	111		R	11	6	0	0	1	1	0	0	26
Thyroid Gland	P		2		0	2	0	0		1	Rectum	P		3		0	2	0	0		0
	R	19	48	0	0	4	0	1	0	28		R									
Parathyroid Gland	P		3		0	2	0	0		1	Kidney	P		2		3	2	1	0		1
	R											R	16	53	0	2	1	1	1	0	15
Adrenal Gland	P		2		0	1	1	0		1	Urinary Bladder	P		2		0	2	0	0		1
	R	45	4	1	0	0	0	0	0	16		R	18	14	3	0	2	2	1	0	27
Appendix	P		2		0	3	0	0		0	Testis	P		2		0	2	0	0		1
	R											R	3	23	7	0	7	2	1	0	11
Bone Marrow	P		3		0	1	1	0		0	Prostate	P		2		0	2	1	0		0
	R											R	16	14	7	0	4	0	3	1	41
Lymph Node	P		2		0	0	1	0		0	Epididymis	P		2		0	1	0	2		1
	R											R									
Tonsil	P		2		0	2	1	0		0	Seminal Vesicle	P		2		0	2	0	0		1
	R											R									
Spleen	P		0		0	0	0	0		0	Fallopian Tube	P		2		0	1	0	0		1
	R	214	3	0	0	5	0	1	0	42		R	19	9	2	0	4	0	5	0	45
Heart Muscle	P		1		0	2	1	0		1	Breast	P		1		0	3	1	0		1
	R	5	4	4	0	3	0	0	0	13		R	18	17	0	0	4	0	2	15	26
Skeletal Muscle	P		1		1	2	0	0		1	Vagina	P		1		0	0	0	0		0
	R	1	2	0	0	0	0	0	0	3		R	16	14	4	0	3	0	1	0	37
Smooth Muscle	P		1		0	0	0	0		1	Cervix, Uterine	P		2		0	1	1	0		1
	R											R	11	10	2	0	3	0	1	0	45
Lung	P		1		0	2	1	0		1	Endometrium	P		1		0	2	1	0		1
	R	57	13	1	0	2	1	1	0	23		R	8	7	0	0	1	0	0	3	47
Nasopharynx	P		2		0	2	0			1	Ovary	P		1		0	1	0	0		0
	R											R	5	2	1	0	1	0	0	1	49
Bronchus	P		3		0	2	0	0		1	Placenta	P		2		0	2	1	0		1
	R											R									
Liver	P		2		0	2	0	0		0	Soft Tissue	P		1		0	0	1	0		1
	R	6	1	0	0	1	1	0	0	3		R									
Gall Bladder	P		2		0	3	0	0		0	Adipose Tissue	P									
	R											R	21	13	0	0	1	0	2	0	20
Pancreas	P		2		3	2	0	0		0	Skin	P		2		0	2	1	0		1
	R	3	11	3	0	0	1	0	0	6		R	5	27	3	0	3	0	0	0	17
Salivary Gland	P		2		0	2	0	0		0	Sum RNA		748	442	51	7	72	12	26	30	1057
	R	14	23	3	1	1	0	2	0	17	Sum Protein		n/a	78	n/a	10	70	23	2	n/a	31

## Supplementary Materials

The following are available online at https://www.mdpi.com/1999-4923/11/9/454/s1.

## Abbreviations

CCN: Cyclin; CCOC: Clear Cell Ovarian Carcinoma; GEO: Gene Expression Omnibus; GMQE: Global Quality Estimation Scores; GPCR: G-Protein Coupled Receptor; HGSOC: High Grade Serous Ovarian Carcinoma; HOSE: Human Ovarian Surface Epithelium; HPA: Human Protein Atlas; LGSOC: Low Grade Serous Ovarian Carcinoma; RMA: Robust Multi-Array.
