# Supplementary material for: Systematic Meta-Analysis Identifies Co-Expressed Kinases and GPCRs in Ovarian Cancer Tissues Revealing a Potential for Targeted Kinase Inhibitor Delivery
Source: Pharmaceutics. 2019 Sep 2;11(9):454. doi: 10.3390/pharmaceutics11090454 (PMC6781325; doi:10.3390/pharmaceutics11090454)

Supplementary information

Information S1: list of kinase and cyclin gene symbols (red: not included in the GLP570 platform)

Kinases

AAK1, AATK, ABL1, ABL2, ACVR1, ACVR1B, ACVR1C, ACVR2A, ACVR2B, ACVRL1, ADCK1, ADCK2, ADCK5, AKT1, AKT2, AKT3, ALK, ALPK1, ALPK2, ALPK3, AMHR2, ANKK1, ARAF, ATM, ATR, AURKA, AURKB, AURKC, AXL, BCKDK, BLK, BMP2K, BMPR1A, BMPR1B, BMPR2, BMX, BRAF, BRSK1, BRSK2, BTK, BUB1, BUB1B, CAMK1, CAMK1D, CAMK1G, CAMK2A, CAMK2B, CAMK2D, CAMK2G, CAMK4, CAMKK1, CAMKK2, CAMKV, CASK, CDC42BPA, CDC42BPB, CDC42BPG, CDC7, CDK1, CDK10, CDK11A, CDK11B, CDK12, CDK13, CDK14, CDK15, CDK16, CDK17, CDK18, CDK19, CDK2, CDK20, CDK3, CDK4, CDK5, CDK6, CDK7, CDK8, CDK9, CDKL1, CDKL2, CDKL3, CDKL4, CDKL5, CHEK1, CHEK2, CHUK, CIT, CLK1, CLK2, CLK3, CLK4, COQ8A, COQ8B, CSF1R, CSK, CSNK1A1, CSNK1A1L, CSNK1D, CSNK1E, CSNK1G1, CSNK1G2, CSNK1G3, CSNK2A1, CSNK2A2, CSNK2A3, DAPK1, DAPK2, DAPK3, DCLK1, DCLK2, DCLK3, DDR1, DDR2, DMPK, DSTYK, DYRK1A, DYRK1B, DYRK2, DYRK3, DYRK4, EEF2K, EGFR, EIF2AK1, EIF2AK2, EIF2AK3, EIF2AK4, EPHA1, EPHA10, EPHA2, EPHA3, EPHA4, EPHA5, EPHA6, EPHA7, EPHA8, EPHB1, EPHB2, EPHB3, EPHB4, EPHB6, ERBB2, ERBB3, ERBB4, ERN1, ERN2, FASTK, FER, FES, FGFR1, FGFR2, FGFR3, FGFR4, FGR, FLT1, FLT3, FLT4, FRK, FYN, GAK, Gm4922, Gm7168, GRK1, GRK2, GRK3, GRK4, GRK5, GRK6, GRK7, GSK3A, GSK3B, HASPIN, HCK, HIPK1, HIPK2, HIPK3, HIPK4, HUNK, ICK, IGF1R, IKBKB, IKBKE, ILK, INSR, INSRR, IRAK1, IRAK2, IRAK3, IRAK4, ITK, JAK1, JAK2, JAK3, KALRN, KDR, KIT, KSR1, KSR2, LATS1, LATS2, LCK, LIMK1, LIMK2, LMTK2, LMTK3, LRRK1, LRRK2, LTK, LYN, MAK, MAP2K1, MAP2K2, MAP2K3, MAP2K4, MAP2K5, MAP2K6, MAP2K7, MAP3K1, MAP3K10, MAP3K11, MAP3K12, MAP3K13, MAP3K14, MAP3K15, MAP3K19, MAP3K2, MAP3K20, MAP3K21, MAP3K3, MAP3K4, MAP3K5, MAP3K6, MAP3K7, MAP3K8, MAP3K9, MAP4K1, MAP4K2, MAP4K3, MAP4K4, MAP4K5, MAPK1, MAPK10, MAPK11, MAPK12, MAPK13, MAPK14, MAPK15, MAPK3, MAPK4, MAPK6, MAPK7, MAPK8, MAPK9, MAPKAPK2, MAPKAPK3, MAPKAPK5, MARK1, MARK2, MARK3, MARK4, MAST1, MAST2, MAST3, MAST4, MASTL, MATK, MELK, MERTK, MET, MINK1, MKNK1, MKNK2, MLKL, MOK, MOS, MST1R, MTOR, MUSK, MYLK, MYLK2, MYLK3, MYLK4, MYO3A, MYO3B, NEK1, NEK10, NEK11, NEK2, NEK3, NEK4, NEK5, NEK6, NEK7, NEK8, NEK9, NIM1K, NLK, NRBP1, NRBP2, NRK, NTRK1, NTRK2, NTRK3, NUAK1, NUAK2, OBSCN, OXSR1, PAK1, PAK2, PAK3, PAK4, PAK5, PAK6, PAN3, PASK, PBK, PDGFRA, PDGFRB, PDIK1L, PDK1, PDK2, PDK3, PDK4, PDPK1, PDPK2P, PEAK1, PHKG1, PHKG2, PIK3CA, PIK3CG, PIK3R4, PIM1, PIM2, PIM3, PINK1, PKDCC, PKMYT1, PKN1, PKN2, PKN3, PLK1, PLK2, PLK3, PLK4, PLK5, PNCK, POMK, PRAG1, PRKAA1, PRKAA2, PRKACA, PRKACB, PRKACG, PRKCA, PRKCB, PRKCD, PRKCE, PRKCG, PRKCH, PRKCI, PRKCQ, PRKCZ, PRKD1, PRKD2, PRKD3, PRKDC, PRKG1, PRKG2, PRKX, PRKY, PRPF4B, PSKH1, PSKH2, PTK2, PTK2B, PTK6, PTK7, PXK, RAF1, RET, RIOK1, RIOK2, RIOK3, RIPK1, RIPK2, RIPK3, RIPK4, RNASEL, ROCK1, ROCK2, ROR1, ROR2, ROS1, RPS6KA1, RPS6KA2, RPS6KA3, RPS6KA4, RPS6KA5, RPS6KA6, RPS6KB1, RPS6KB2, RPS6KC1, RPS6KL1, RYK, SBK1, SBK2, SBK3, SCYL1, SCYL2, SCYL3, SGK1, SGK2, SGK3, SGK494, SIK1, SIK2, SIK3, SLK, SMG1, Smok1, Smok2a, Smok2b, Smok3a, Smok3b, Smok4a, Smoktcr, SNRK, SPEG, SRC, SRMS, SRPK1, SRPK2, SRPK3, STK10, STK11, STK16, STK17A, STK17B, STK24, STK25, STK26, STK3, STK31, STK32A, STK32B, STK32C, STK33, STK35, STK36, STK38, STK38L, STK39, STK4, STK40, STKLD1, Stk-ps2, STRADA, STRADB, STYK1, SYK, TAOK1, TAOK2, TAOK3, TBCK, TBK1, TEC, TEK, TESK1, TESK2, TEX14, TGFBR1, TGFBR2, TIE1, TLK1, TLK2, TNIK, TNK1, TNK2, TNNI3K, TP53RK, TRIB1, TRIB2, TRIB3, TRIO, TRPM6, TRPM7, TSSK1B, TSSK2, TSSK3, TSSK4, Tssk5, TSSK6, TTBK1, TTBK2, TTK, TTN, TXK, TYK2, TYRO3, UHMK1, ULK1, ULK2, ULK3, ULK4, VRK1, VRK2, VRK3, WEE1, WEE2, WNK1, WNK2, WNK3, WNK4, YES1, ZAP70

Cyclins

CCNA1, CCNA2, CCNB1, CCNB2, CCNB3, CCNC, CCND1, CCND2, CCND3, CCNE1, CCNE2, CCNF, CCNG1, CCNG2, CCNH, CCNI, CCNI2, CCNJ, CCNJL, CCNK, CCNL1, CCNL2, CCNO, CCNQ, CCNT1, CCNT2, CCNY, CCNYL1, CCNYL2, CCNYL3, CDK5R1, CDK5R2

Figure S2 (pages 2-6): Heat maps and whisker plots for GSE10971, GSE14401, GSE14407, GSE18520, GSE27651, GSE29450, GSE52037, GSE54388 and GSE105437.

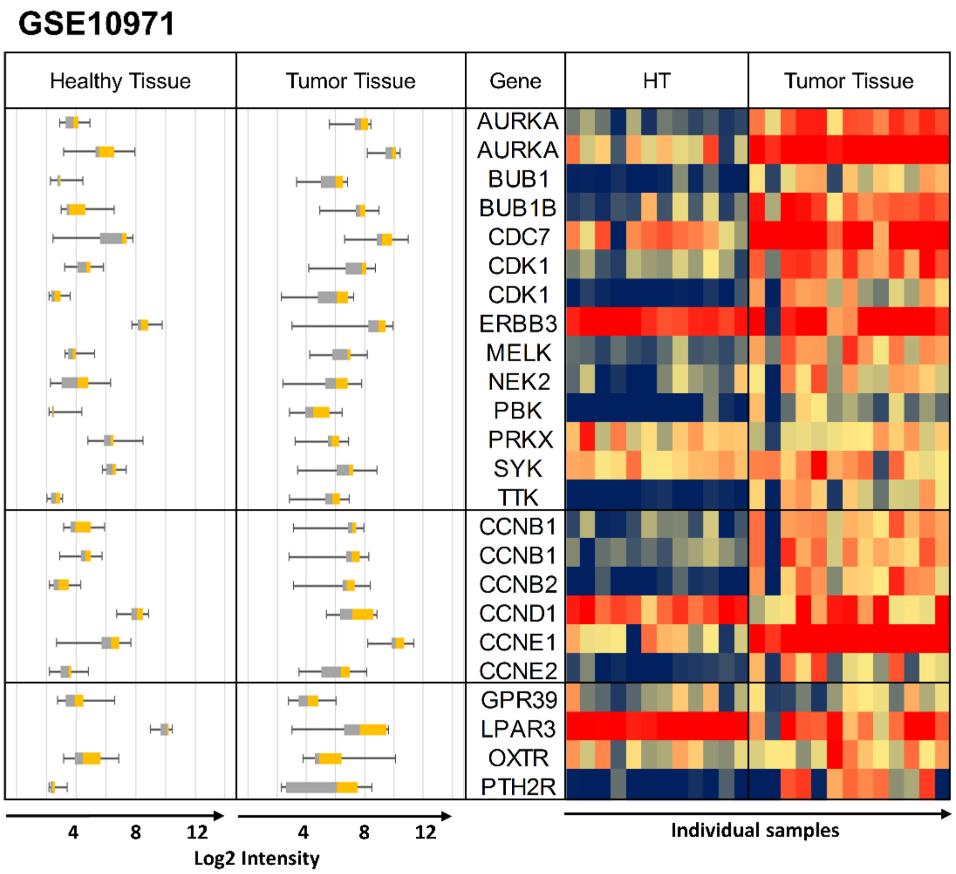

GSE14401

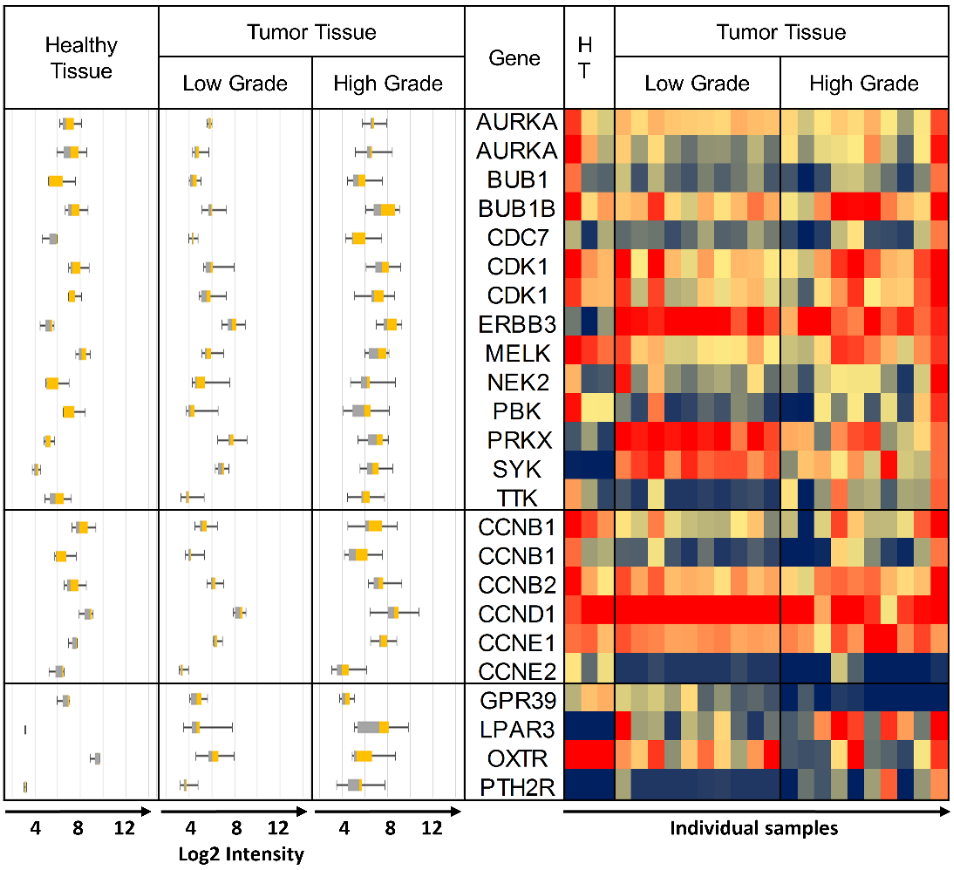

GSE14407

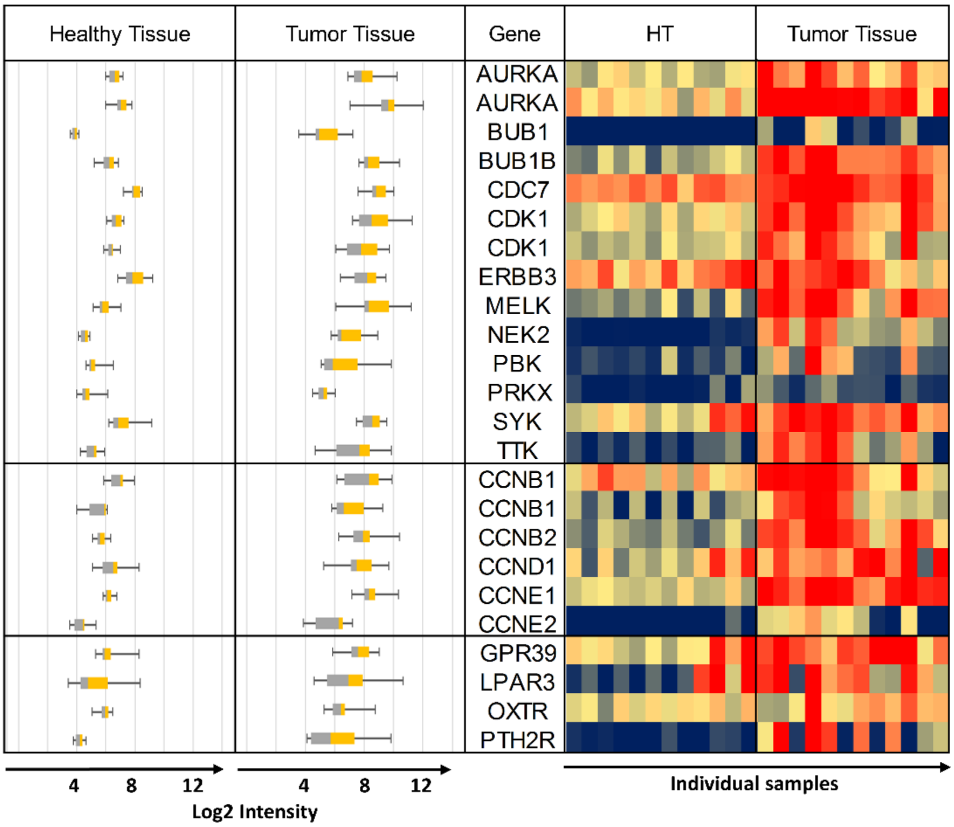

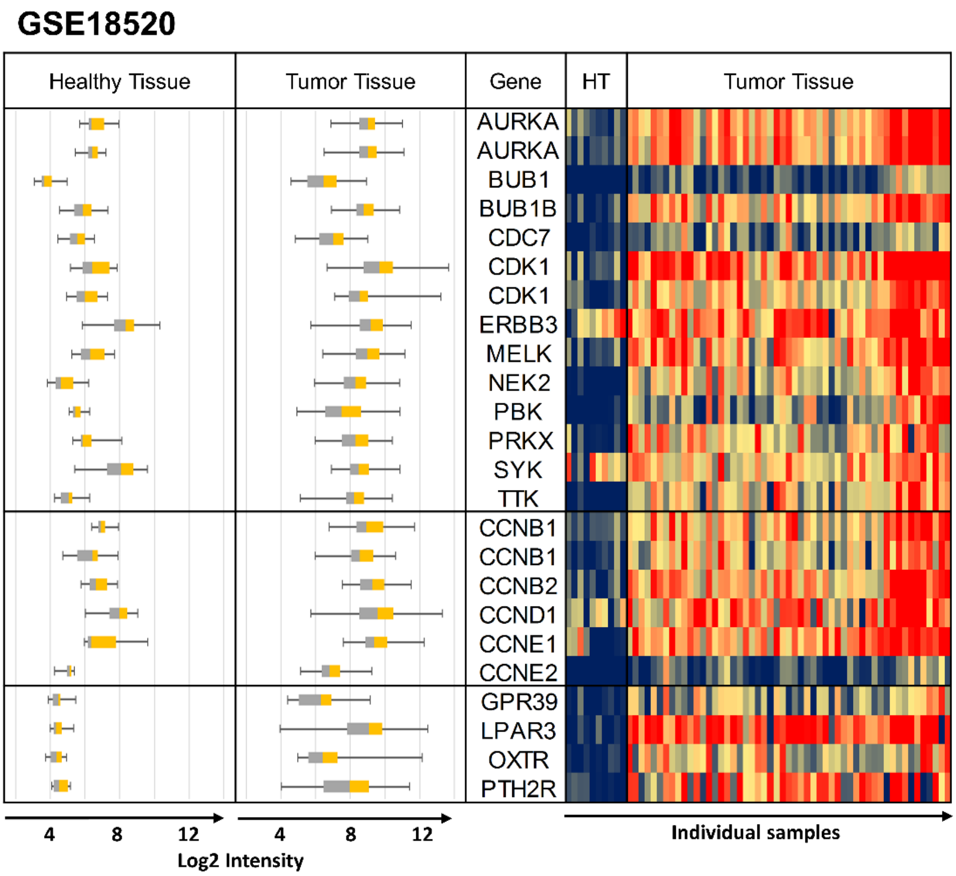
GSE18520
Healthy Tissue
Tumor Tissue
Gene
HT
Tumor Tissue
AURKA
AURKA
BUB1
BUB1B
CDC7
CDK1
CDK1
ERBB3
MELK
NEK2
PBK
PRKX
SYK
TTK
CCNB1
CCNB1
CCNB2
CCND1
CCNE1
CCNE2
GPR39
LPAR3
OXTR
PTH2R
4 8 12
4 8 12
Log2 Intensity
Individual samples

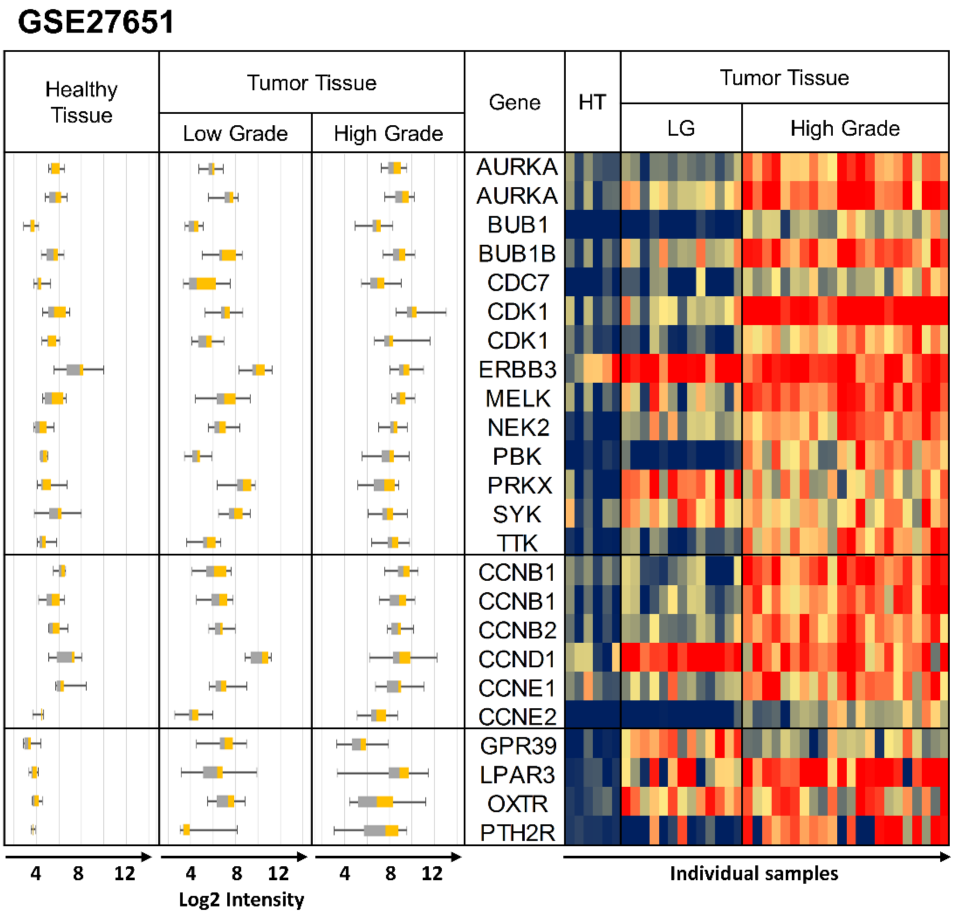
GSE27651
Healthy Tissue
Tumor Tissue
Low Grade
High Grade
Gene
HT
Tumor Tissue
LG
High Grade
AURKA
AURKA
BUB1
BUB1B
CDC7
CDK1
CDK1
ERBB3
MELK
NEK2
PBK
PRKX
SYK
TTK
CCNB1
CCNB1
CCNB2
CCND1
CCNE1
CCNE2
GPR39
LPAR3
OXTR
PTH2R
4 8 12
4 8 12
4 8 12
Log2 Intensity
Individual samples

GSE29450

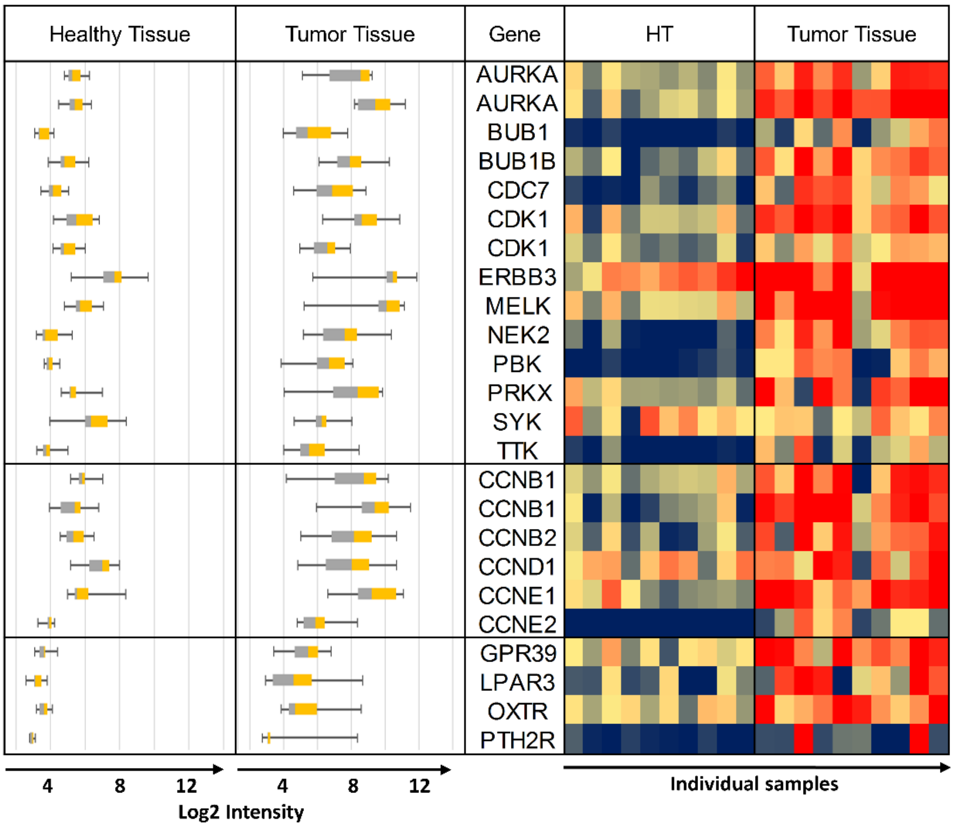

GSE52037

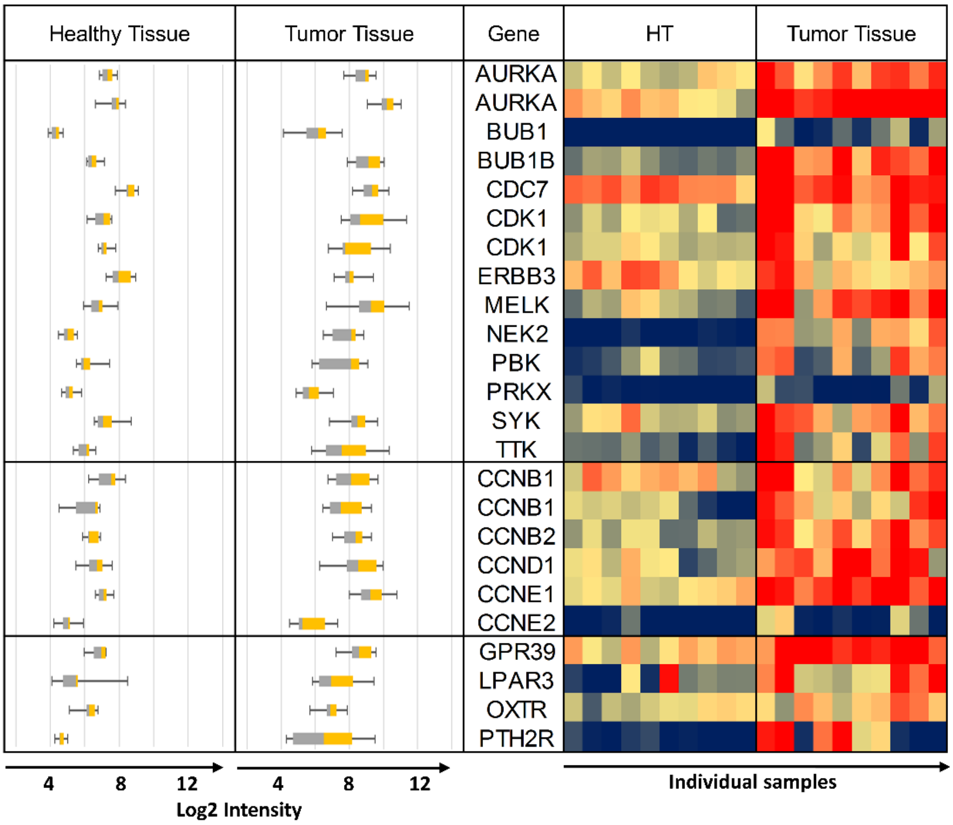

GSE54388

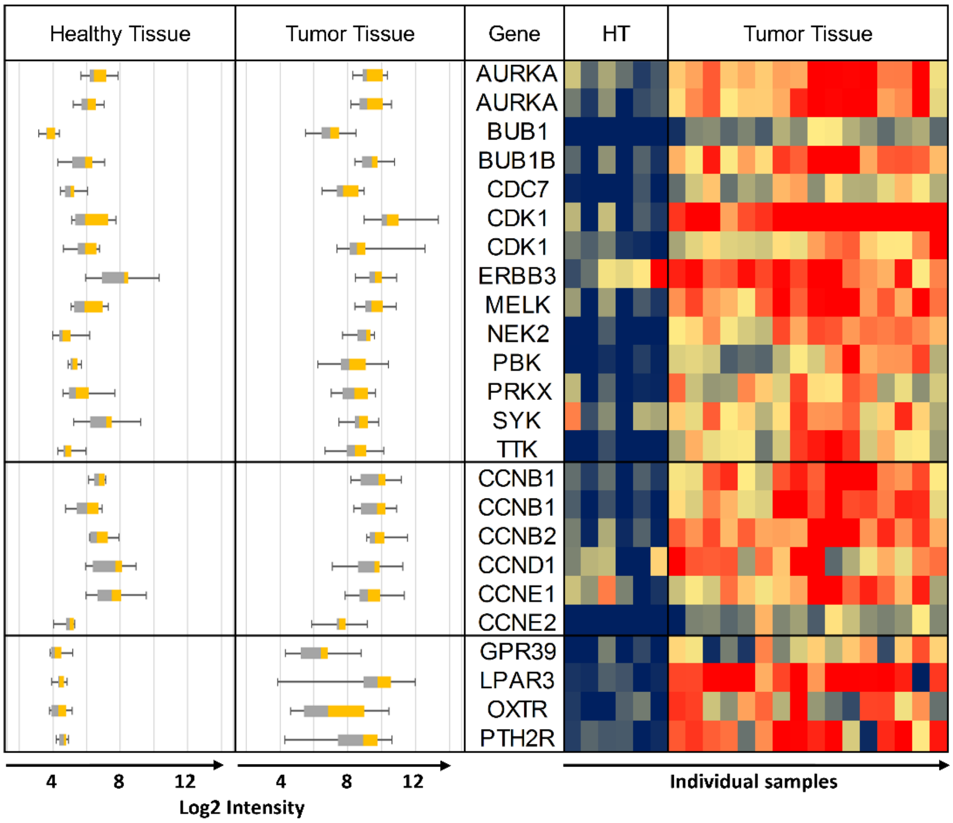

GSE105437

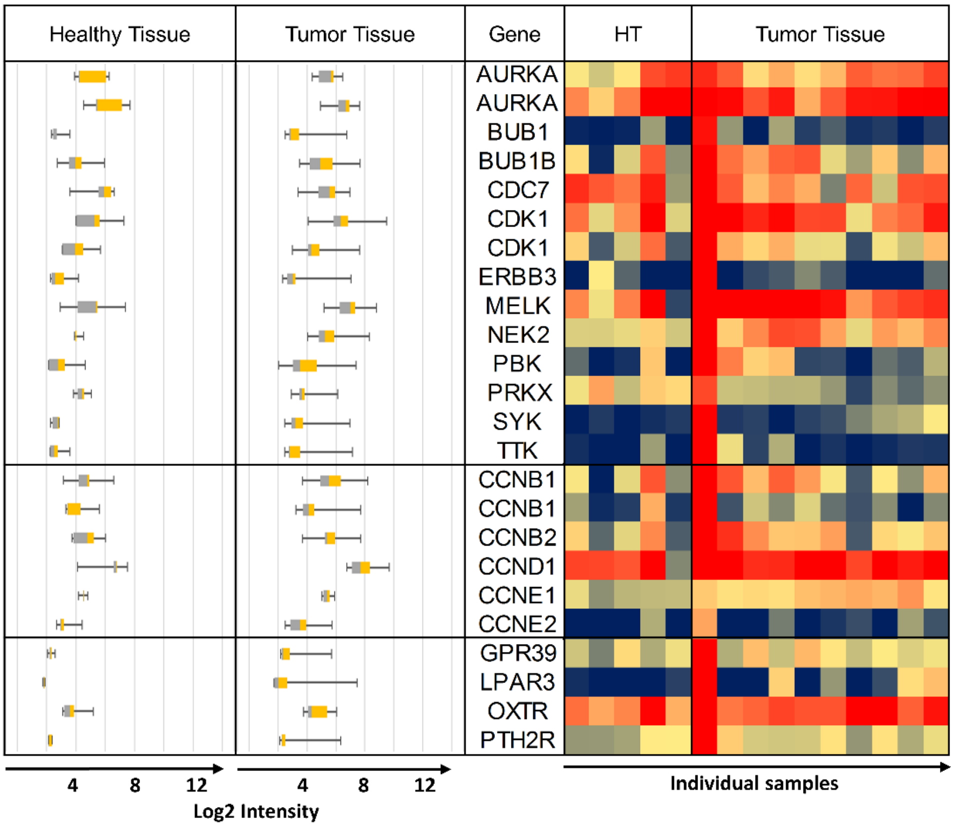

Supplement: Supplementary file 1 [file pharmaceutics-11-00454-s001.pdf]
